# Underlying cardiopulmonary conditions as a risk factor for influenza and respiratory syncytial virus infection among community‐dwelling adults aged ≥ 65 years in Thailand: Findings from a two‐year prospective cohort study

**DOI:** 10.1111/irv.12855

**Published:** 2021-03-25

**Authors:** Prabda Praphasiri, Manash Shrestha, Jayanton Patumanond, Sutthichai Nakphook, Sutthinan Chawalchitiporn, Darunee Ditsungnoen, Fatimah S. Dawood, Joshua A. Mott, Kriengkrai Prasert

**Affiliations:** ^1^ Influenza Program Thailand MOPH‐US CDC Collaboration Nonthaburi Thailand; ^2^ Mahidol University Nakhon Pathom Thailand; ^3^ Chiang Mai University Chiang Mai Thailand; ^4^ Institute of Preventive Medicine Department of Disease Control Ministry of Public Health Nonthaburi Thailand; ^5^ Nakhon Phanom Provincial Hospital Nakhon Phanom Thailand; ^6^ Influenza Division US Centers for Disease Control and Prevention Atlanta GA USA

**Keywords:** cardiopulmonary conditions, influenza, older adult, respiratory syncytial virus, Thailand

## Abstract

**Background:**

Adults with cardiopulmonary conditions may be at increased risk of influenza and respiratory syncytial virus (RSV) infection, but it is not well‐established and few data are available from middle‐income countries.

**Methods:**

Using data from a prospective cohort study of influenza vaccine effectiveness, we estimated and compared the incidences of influenza and RSV between community‐dwelling Thai adults aged ≥ 65 years with and without cardiopulmonary conditions. During May 2015‐May 2017, older adults in a rural province in Thailand were followed‐up with weekly surveillance for acute respiratory illness (ARI), defined broadly as new onset or worsening of cough with or without fever, and hospitalized ARI. When ill, nasal self‐swabs and/or nasopharyngeal swabs were collected for reverse‐transcription polymerase chain reaction testing. We used Poisson regression to calculate incidence rate ratios (IRR), adjusting for age, sex, current smoking, number of hospital visits, weekly influenza activity, and influenza vaccination.

**Results:**

Overall, 3220 adults with a median age of 71 years (IQR 68‐76) were enrolled; 1324 (41.1%) were male; and 313 (9.7%) had ≥1 underlying cardiopulmonary condition, most commonly chronic obstructive pulmonary disease (131; 41.2%) or asthma (73; 23.3%). Participants with cardiopulmonary conditions had higher incidences of ARI, influenza, and RSV than those without (Adjusted IRR: 1.84, 95% CI 1.64‐2.07; 1.86, 95% CI 1.07‐3.26; 2.04, 95% CI 1.11‐3.76, respectively).

**Conclusion:**

Older adults in rural Thailand with cardiopulmonary conditions have increased rates of ARI, influenza, and RSV infections. Our findings support efforts to ensure this population has access to influenza vaccines and other respiratory illness prevention measures.

## 1 INTRODUCTION

Influenza and respiratory syncytial virus (RSV) are respiratory pathogens that cause a substantial disease burden among adults aged 65 years and above.^1^ Global estimates suggest that approximately two‐thirds of all seasonal influenza‐related mortalities occur among older adults with more than 260 000 deaths annually.^2^ Data from developed countries show that respiratory illnesses due to RSV among older adults may exceed one million episodes and cause around 14 000 in‐hospital deaths every year.^3^ Older adults with cardiopulmonary conditions are at an increased risk of severe illness due to influenza and RSV infection.^4^, ^5^ However, it is not fully established whether cardiopulmonary conditions present an increased risk for influenza and RSV infections, and there is little data on the burden of infection from these pathogens among persons with cardiopulmonary conditions in middle‐income countries.

Thailand is an upper‐middle‐income country in tropical Southeast Asia, where recognition of influenza and RSV disease burden is growing. Since 2008, adults aged ≥ 65 years and those with chronic diseases have been recommended for annual influenza vaccination by the Thai Ministry of Public Health (MoPH).^6^ Nonetheless, influenza vaccine uptake among older adults is still less than 20% (Bureau of General Communicable Diseases, Thailand MoPH). Studies among hospitalized patients suggest that the prevalence of RSV‐associated respiratory illnesses in Thailand is around 2%‐9% among older adults.^7^, ^8^, ^9^, ^10^, ^11^ However, reliable estimates of infection rates in the community remain limited. Using data from a longitudinal prospective cohort study of influenza vaccine effectiveness among rural community‐dwelling Thai adults aged ≥ 65 years,^12^ we compared the incidence of influenza and RSV in those with and without cardiopulmonary conditions.

## 2 METHODS

### 2.1 Study design

The details of this study have been described previously.^12^ In brief, a prospective cohort of 3500 adults aged ≥ 65 years was recruited by systematic random sampling in two districts of Nakhon Phanom province, Thailand from May 2015 and followed up for 2 years until May 2017. Prior to the start of the study, a targeted campaign was organized at the study sites by the Thai MoPH to enhance influenza vaccine uptake among older adults through mobile clinics and increased vaccine supply.^13^ Individuals were not eligible to participate in the cohort if they resided in a long term care facility, did not reside in the community for at least a year, were unable to communicate with study staff, were unable/unwilling to provide nasal self‐swabs, or had an acute medical condition or tumor in or near the nose that would preclude nasal swab collection. Trained health volunteers collected baseline data and followed the participants with weekly telephone surveillance for acute respiratory illness (ARI). Participants were asked to self‐collect anterior nasal swabs for each ARI episode as previously validated.^14^ For participants who visited health centers/hospitals for their illnesses, nasopharyngeal swabs were collected by research nurses on site. The number of hospital visits of the participants (for ARI or other reasons) during the study period was obtained from the district and provincial hospital records.

### 2.2 Definitions

Acute respiratory illness (ARI) was defined as a new onset of cough or worsening of a chronic cough. Severe ARI (SARI) was defined as an illness requiring hospitalization with a measured axillary temperature ≥38°C plus a new onset of cough, worsening of a chronic cough, or difficulty in breathing. Cardiopulmonary condition was defined as the presence of any known physician‐diagnosed chronic cardiovascular conditions (such as coronary artery diseases, cardiac arrhythmias, valvular heart diseases, cardiomyopathy) and/or any chronic lung diseases (such as chronic obstructive pulmonary disease (COPD), asthma, chronic bronchitis, pulmonary tuberculosis, emphysema, and lung fibrosis).

### 2.3 Specimen collection, storage, laboratory testing

All respiratory specimens (including participant‐collected and nurse‐collected swabs) were transported to refrigerators at sub‐district health centers within 24 hours of collection, kept between 2‐8°C before being frozen in liquid nitrogen tanks below −70°C, and sent weekly to the Thai National Institute of Health (NIH) in Nonthaburi. Respiratory specimens were tested for influenza viruses and RSV with real‐time reverse‐transcription polymerase chain reaction test (rRT‐PCR) according to CDC/WHO protocols.^15^, ^16^

### 2.4 Data analysis

We calculated the incidence per 1000 person‐years of laboratory‐confirmed influenza and RSV‐associated ARI and SARI by dividing the total episodes by total person‐time under observation. Weeks of intermittent loss to follow‐up (due to missed swab collection and/or missed surveillance contacts) were excluded from the person‐time denominator when calculating the incidence rates. Multivariable Poisson regression was used to calculate incidence rate ratios (IRR) between participants with and without any underlying cardiopulmonary conditions, adjusting for age, sex, current smoking status, influenza vaccination status (participants receiving influenza vaccine in at least one of the two study years were considered as vaccinated), and weekly influenza activity (influenza infections detected by weekly NIH surveillance, categorized into quartiles). As participants with a better healthcare‐seeking behavior may visit the hospitals more and potentially acquire and get tested for respiratory infections more than those who do not visit the hospitals often, the number of hospital visits during the study years was also adjusted in the regression analysis. All data analyses were conducted using STATA software version 14.2 (StataCorp LP, College Station, TX, USA).

### 2.5 Ethical considerations

Written informed consent was obtained from all participants and the study was approved by the Ethical Review Committee, Department of Disease Control, MoPH (Nonthaburi, Thailand). The US Centers for Disease Control and Prevention (Atlanta, Georgia) relied on this committee for ethical approval.

## 3 RESULTS

### 3.1 Participant characteristics

Of 3500 participants screened, 3287 were eligible, and 3220 adults were enrolled in the study. The participants had a median age of 71 years (interquartile range [IQR] 68‐76) and 1324 (41%) were male (Table 1). The proportion of cohort participants that were vaccinated for influenza was 52% (1666/3220) in 2015 and 48% (1498/3123) in 2016. In total, 313 (9.7%) had one or more underlying cardiopulmonary condition, most commonly COPD (131; 41.2%), asthma (73; 23.3%), or chronic bronchitis (68; 21.7%) (Table 2). The participants with cardiopulmonary conditions were more likely to be older, male, current smokers, have more other co‐morbidities, and have a higher number of hospital visits than those without cardiopulmonary conditions (all *P* < .05; Table 1).

**TABLE 1:** Participant characteristics in a population‐based cohort study, Nakhon Phanom Province, Thailand, May 2015‐May 2017

Characteristics	Cohort participants		*P*‐value
	Without cardiopulmonary conditions (n = 2907) n (%)	With cardiopulmonary conditions (n = 313) n (%)	
Total person‐years	5532	558	
Age (years), median (IQR)	72 (69, 76)	73 (70, 78)	.003
Age group			
<75 years	1925 (66.2)	190 (60.7)	.052
≥75 years	982 (33.8)	123 (39.3)	
Male	1157 (39.8)	167 (53.4)	<.001
Current smokers	954 (32.8)	142 (45.4)	<.001
Influenza vaccination uptake			
2015‐2016	1497 (51.5)	169 (54.0)	.405
2016‐2017	1347 (47.5)	151 (53.0)	.082
Co‐morbidity			
Yes	1163 (40.0)	156 (49.8)	<.001
Hypertension	670 (23.1)	85 (27.2)	.107
Diabetes	543 (18.7)	62 (19.8)	.648
Neuromuscular diseases	83 (2.9)	14 (4.5)	.117
Chronic kidney diseases	79 (2.7)	11 (3.5)	.370
Cerebrovascular diseases	74 (2.6)	11 (3.5)	.350
Chronic liver diseases	13 (0.5)	3 (1.0)	.199
Thalassemia	4 (0.1)	6 (1.9)	<.001
Cancer	81 (2.8)	17 (5.4)	.015
Chemotherapy	10 (0.3)	2 (0.6)	.328
Mean number of hospital visits (SD)	2.8 (1.6)	3.5 (1.1)	<.001

Abbreviations: IQR, interquartile range; n, number.

**TABLE 2:** Cardiopulmonary conditions among participants in a population‐based cohort study, Nakhon Phanom Province, Thailand, May 2015‐May 2017

Cardiopulmonary condition^a^	Total = 313
	n (%)
1. Cardiovascular diseases	
Coronary artery diseases	48 (15.3)
Cardiac arrhythmias	27 (8.6)
Heart valve diseases	10 (3.2)
Cardiomyopathy	3 (1.0)
2. Chronic lung diseases	
Chronic obstructive pulmonary disease	131 (41.2)
Asthma	73 (23.3)
Chronic bronchitis	68 (21.7)
Pulmonary tuberculosis	39 (12.5)
Emphysema	1 (0.3)
Lung fibrosis	1 (0.3)

^a^ Each participant could have more than one cardiopulmonary condition.

The participants were followed up for a total of 316 690 person‐weeks (29 005 person‐weeks for participants with cardiopulmonary conditions and 287 685 person‐weeks for participants without cardiopulmonary conditions). During the 2‐year follow‐up, two participants withdrew and 200 (6.2%) died. Overall, 2365 ARI/SARI episodes (2296 ARI and 69 SARI) were reported in the cohort of which 2362 (99.8%) respiratory swabs were collected (1969 nasal self‐swabs and 393 nasopharyngeal swabs by research nurses).

### 3.2 Incidence of influenza and RSV

**FIGURE 1:**
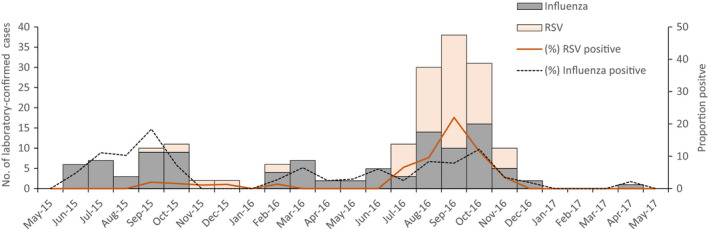
Number of laboratory‐confirmed influenza and RSV infection and proportion of positive cases by month among older adults aged ≥ 65 years in population‐based cohort study, Nakhon Phanom province, Thailand, May 2015‐May 2017

Influenza A(H3N2) was the predominant circulating influenza virus in both years. The median time from symptom onset to collection of nasal swabs was 1 day (IQR 1‐2) and nasopharyngeal swabs were 2 days (IQR 1‐3), which were not different among participants with or without cardiopulmonary conditions (*P*‐values .589 and .746, respectively). Overall, 105 (3.3%) cohort participants had an influenza virus infection (influenza A(H1N1)=17; A(H3N2)=74; B = 14) and 81 (2.5%) had an RSV infection. No participant experienced dual infections from both influenza and RSV. Laboratory‐confirmed influenza infections were detected most in the first year in September (18.4%), followed by October in the second year (12.2%); most RSV infections were detected during August to October in the second year, with the peak in September at 22.0% (Figure 1).

Participants with cardiopulmonary conditions had a higher incidence of ARI (645/1000 person‐years vs 350/1000 person‐years, *P* < .001) and SARI (87.8/1000 vs 3.6/1000, *P* < .001) than those without. Adjusted IRR for laboratory‐confirmed RSV, all influenza, influenza A, and influenza B viruses were 2.04 (95% confidence interval [CI] 1.11‐3.76), 1.86 (95% CI 1.05‐3.26), 1.84 (95% CI 1.01‐3.36), and 2.04 (95% CI 0.44‐9.57), respectively (Table 3). The seasonal trend of RSV, influenza, and ARI was generally similar among participants with and without cardiopulmonary conditions with higher incidence observed in the second year, particularly during August‐November (Supplemental Figure S1). Among the participants with SARI, there were only two influenza and six RSV‐confirmed hospitalization episodes. Although there were 200 deaths throughout the study period, only one participant (without cardiopulmonary condition) died following an RSV‐confirmed SARI.

**TABLE 3:** Incidence rate ratio of Respiratory Syncytial Virus, Influenza A and B among participants with or without cardiopulmonary conditions

	Participants without CPC		Participants with CPC		Crude IRR (95% CI)	Adjusted IRR^d^ (95% CI)
No. of person	2907		313			
Person‐years	5532		558			
Endpoints	No.	Rate^a^ (95% CI)	No.	Rate ^a^ (95% CI)		
ARI^b^	1936	350 (337, 363)	360	645 (604, 685)	1.84 (1.64, 2.06)*	1.84 (1.64, 2.07)*
SARI^c^	20	3.6 (2.2, 5.6)	49	87.8 (65.7, 114.4)	24.30 (14.18, 43.16)*	24.56 (14.05, 42.95)*
RSV infection	68	12.3 (9.6, 15.6)	13	23.2 (12.5, 39.5)	1.90 (0.96, 3.46)*	2.04 (1.11, 3.76)*
Influenza	90	16.3 (13.1, 20.0)	15	26.9 (15.1, 44.0)	1.65 (0.89, 2.87)	1.86 (1.07, 3.26)*
Influenza A	78	14.1 (9.9, 16.9)	13	23.3 (12.5, 39.5)	1.65 (0.84, 2.99)	1.83 (1.01, 3.36)*
Influenza B	12	2.2 (1.1, 3.9)	2	3.6 (0.4, 12.9)	1.65 (0.18, 7.43)	2.04 (0.44, 9.57)

Abbreviations: ARI, acute respiratory illness; CI, confidence interval; CPC, cardiopulmonary conditions; IRR, incidence rate ratio; No., Number; RSV, Respiratory syncytial virus; SARI, severe acute respiratory illness.

^a^ Incidence rate / 1000 person‐years.

^b^ ARI is defined as a new onset of cough or worsening of chronic cough, with or without fever.

^c^ SARI is defined as new onset of cough, worsening of chronic cough or difficulty breathing with a fever ≥ 38.0°C that required hospitalization.

^d^ Poisson regression analysis is adjusted for age, sex, current smoking, influenza vaccination, weekly influenza activity, and number of hospital visits.

* Statistically significant at *P*‐value < .05.

## 4 DISCUSSION

Among a 2‐year prospective cohort study of >3000 community‐dwelling adults in Thailand, around 10% of participants had underlying cardiopulmonary conditions, and cardiopulmonary conditions conferred increased rates of all‐cause ARI, influenza A, and RSV infections. These findings provide additional support for influenza vaccination recommendations for persons with underlying cardiopulmonary conditions and add to the limited body of evidence about influenza and RSV disease burden among older adults in middle‐income countries.

The seasonality of influenza and RSV was similar to the pattern identified in past studies in which the infections peaked during the rainy months of June to October.^10^, ^17^, ^18^ Although a previous study reported a year‐round transmission of influenza with two peaks, one in February and one in September,^17^ we could not detect influenza among the participants in some months and the peak in February was missing in the second year of the study. As for RSV, the bulk of the infections were observed only in the second year which overlapped notably with the August‐November peak of influenza among both groups of participants—with and without cardiopulmonary conditions. The pattern of RSV transmission during the rainy season is in alignment with studies from different tropical countries and may guide the timing of future RSV vaccination programs.^17^, ^19^

Incidences of influenza and RSV are higher in our study than in previous studies.^7^, ^8^, ^10^ This may be partly due to a more sensitive case definition of ARI. Since many older adults with influenza and RSV can present without fever, we used a definition of ARI without any body temperature criteria.^12^ This might have resulted in the inclusion of more illness episodes with laboratory confirmation than in the past studies which required either fever or hypothermia (<35.5°C) to trigger swab collection. Moreover, previous studies primarily assessed hospitalized patients and may not have fully accounted for non‐medically attended cases in the community, even after adjustments in their analyses.^7^, ^8^ In our study, more than 80% of the respiratory specimens were nasal self‐swabs collected by the participants at their homes and only a small proportion of ARI were severe enough to warrant hospitalization, signifying a majority of non‐medically attended cases in the community.

Cardiopulmonary conditions are recognized risk factors for severe outcomes of influenza and RSV infections, especially among older adults.^4^, ^5^ Prior studies have demonstrated that a disproportionate number of hospitalizations for influenza and RSV occur in patients with cardiopulmonary conditions, particularly COPD.^9^, ^10^, ^18^ Our findings suggest that these conditions may themselves be predisposing risk factors for the acquisition of influenza and RSV infections in the community, possibly by affecting immunoregulatory functions in older adults.^19^ Our findings are consistent with evidence from Western countries that documents higher detection of RSV among older adults with underlying cardiopulmonary diseases compared to those without these conditions.^20^, ^21^

This study was conducted in Nakhon Phanom, a rural northeastern province of Thailand bordering Laos, which facilitated comparisons with different previous studies from the province.^7^, ^8^, ^10^ However, the results of this study may not be readily generalizable to other regions of Thailand, especially considering the higher uptake of influenza vaccines of around 50% among the study sample compared to the national estimate of less than 20%. Coupled with vaccine effectiveness of 50% (95% CI 12%‐71%) in the second year of the study,^12^ the higher vaccine uptake may have contributed to a lower incidence of influenza among the participants. Furthermore, the prevalence of COPD observed in our study (4.1%; 131/3220) was relatively lower than those reported among older adults in other regions of Thailand which ranged between 8.0%‐13.4%.^22^, ^23^, ^24^ Incidences of influenza and RSV may likely be higher in other provinces where the vaccine uptake is lower and the prevalence of COPD is higher. Therefore, while Thailand has a national policy of recommending influenza vaccines to older adults and those with chronic diseases,^6^ it is imperative to increase the access to influenza vaccines of adults ≥ 65 years with cardiopulmonary conditions. Our findings also provide empirical support for RSV vaccinations among the older adults with cardiopulmonary conditions in Thailand, when the vaccines being developed against RSV eventually become available for public use.

There are a few other limitations to our study. First, we ascertained cardiopulmonary conditions by participant report alone which may be susceptible to some recall and measurement bias. Second, we did not collect data about exposure to children, household crowding, socioeconomic status, and baseline influenza and RSV antibody titers which may be confounders in the relationship between cardiopulmonary conditions and acquisition/transmission of influenza virus and RSV infection.^20^ Third, a previous analysis comparing participant‐collected nasal swabs to nurse‐collected nasopharyngeal swabs among older adults in Thailand found that participant‐collected nasal swabs were only 78% sensitive for the detection of influenza viruses.^14^ Therefore, our calculation of influenza and RSV incidence may be underestimated. Nevertheless, the strengths of this research lie in the population‐based multiple‐year prospective cohort study design with large sample size, use of a broad case definition of ARI, capture of non‐medically attended ARI, active weekly follow‐up of participants, and laboratory confirmation of the infection outcomes.

In conclusion, cardiopulmonary conditions conferred an additional risk for acquisition of influenza and RSV infections among community‐dwelling older Thai adults aged ≥ 65 years. Despite efforts to enhance influenza vaccine uptake among this cohort as part of the study design, only half of the participants received influenza vaccines during each of the study years. More efforts are needed to ensure adults aged ≥ 65 years, especially those with cardiopulmonary conditions, have access to influenza vaccines and other prevention measures as effective vaccines against RSV are not currently available.

## 5 DISCLAIMER

The findings and conclusions in this report are those of the authors and do not necessarily represent the views of the Centers for Disease Control and Prevention.

## CONFLICT OF INTEREST

The authors declare that they have no personal or financial relationship that could lead to a conflict of interest.

## AUTHOR CONTRIBUTION

**Prabda Praphasiri:** Conceptualization (lead); Data curation (supporting); Investigation (equal); Methodology (equal); Project administration (supporting); Writing‐original draft (equal); Writing‐review & editing (equal). **Manash Shrestha:** Conceptualization (supporting); Formal analysis (supporting); Visualization (supporting); Writing‐original draft (equal); Writing‐review & editing (equal). **Jayanton Patumanond:** Data curation (supporting); Formal analysis (equal); Methodology (equal); Visualization (supporting); Writing‐review & editing (supporting). **Sutthichai Nakphook:** Data curation (supporting); Investigation (supporting); Project administration (supporting); Visualization (supporting); Writing‐review & editing (supporting). **Sutthinan Chawalchitiporn:** Data curation (supporting); Investigation (equal); Project administration (supporting); Writing‐review & editing (supporting). **Darunee Ditsungnoen:** Data curation (supporting); Investigation (equal); Project administration (supporting); Writing‐review & editing (supporting). **Fatimah S Dawood:** Conceptualization (supporting); Methodology (supporting); Supervision (supporting); Validation (equal); Writing‐review & editing (equal). **Joshua Mott:** Conceptualization (supporting); Supervision (lead); Validation (equal); Writing‐review & editing (equal). **Kriengkrai Prasert:** Conceptualization (supporting); Data curation (lead); Formal analysis (equal); Funding acquisition (lead); Investigation (equal); Methodology (equal); Project administration (lead); Visualization (lead); Writing‐review & editing (supporting).

### PEER REVIEW

The peer review history for this article is available at https://publons.com/publon/10.1111/irv.12855.

## Supporting information

Fig S1

## Data Availability

All relevant data are within the paper and the supporting files.

## References

[1] Elliot AJ, Fleming DM. Influenza and respiratory syncytial virus in the elderly. Expert Rev Vaccines. 2008;7(2):249‐258. 18324893 10.1586/14760584.7.2.249

[2] Paget J, Spreeuwenberg P, Charu V, et al. Global mortality associated with seasonal influenza epidemics: new burden estimates and predictors from the GLaMOR Project. J Glob Health. 2019;9(2):020421. 10.7189/jogh.09.020421 PMC6815659 31673337

[3] Shi T, Denouel A, Tietjen AK, et al. Global disease burden estimates of respiratory syncytial virus–associated acute respiratory infection in older adults in 2015: a systematic review and meta‐analysis. J Infect Dis. 2020;222(Supplement_7):S577‐S583. 30880339 10.1093/infdis/jiz059

[4] Grohskopf LA, Sokolow LZ, Broder KR, et al. Prevention and control of seasonal influenza with vaccines: recommendations of the advisory committee on immunization practices ‐ United States, 2017–18 influenza season. MMWR Recomm Rep. 2017;66(2):1‐20. 10.15585/mmwr.rr6602a1 PMC5837399 28841201

[5] Falsey AR, Walsh EE, Esser MT, Shoemaker K, Yu L, Griffin MP. Respiratory syncytial virus–associated illness in adults with advanced chronic obstructive pulmonary disease and/or congestive heart failure. J Med Virol. 2019;91(1):65‐71. 30132922 10.1002/jmv.25285 PMC6900175

[6] Owusu JT, Prapasiri P, Ditsungnoen D, et al. Seasonal influenza vaccine coverage among high‐risk populations in Thailand, 2010–2012. Vaccine. 2015;33(5):742‐747. 25454853 10.1016/j.vaccine.2014.10.029 PMC4610807

[7] Fry AM, Chittaganpitch M, Baggett HC, et al. The burden of hospitalized lower respiratory tract infection due to respiratory syncytial virus in rural Thailand. PLoS One. 2010;5(11):e15098. 21152047 10.1371/journal.pone.0015098 PMC2994907

[8] Olsen SJ, Thamthitiwat S, Chantra S, et al. Incidence of respiratory pathogens in persons hospitalized with pneumonia in two provinces in Thailand. Epidemiol Infect. 2010;138(12):1811‐1822. 20353622 10.1017/S0950268810000646

[9] Hara K, Yahara K, Gotoh K, et al. Clinical study concerning the relationship between community‐acquired pneumonia and viral infection in northern Thailand. Intern Med. 2011;50(9):991‐998. 21532221 10.2169/internalmedicine.50.4738

[10] Naorat S, Chittaganpitch M, Thamthitiwat S, et al. Hospitalizations for acute lower respiratory tract infection due to respiratory syncytial virus in Thailand, 2008‐2011. J Infect Dis. 2013;208(suppl 3):S238‐S245. 24265483 10.1093/infdis/jit456

[11] Thongpan I, Suntronwong N, Vichaiwattana P, Wanlapakorn N, Vongpunsawad S, Poovorawan Y. Respiratory syncytial virus, human metapneumovirus, and influenza virus infection in Bangkok, 2016‐2017. PeerJ. 2019;7:e6748‐e. 30997293 10.7717/peerj.6748 PMC6462397

[12] Prasert K, Patumanond J, Praphasiri P, et al. Effectiveness of trivalent inactivated influenza vaccine among community‐dwelling older adults in Thailand: a two‐year prospective cohort study. Vaccine. 2019;37(6):783‐791. 30616956 10.1016/j.vaccine.2018.12.047

[13] Praphasiri P, Ditsungnoen D, Sirilak S, et al. Predictors of seasonal influenza vaccination among older adults in Thailand. PLoS One. 2017;12(11):e0188422. 29186159 10.1371/journal.pone.0188422 PMC5706686

[14] Goyal S, Prasert K, Praphasiri P, et al. The acceptability and validity of self‐collected nasal swabs for detection of influenza virus infection among older adults in Thailand. Influenza Other Resp. 2017;11(5):412‐417. 10.1111/irv.12471 PMC5596524 28741903

[15] World Health Organization . WHO Information for the Molecular Detection of Influenza Viruses. Geneva, Switzerland: World Health Organization; 2017. http://www.whoint/influenza/gisrs_laboratory/molecular_diagnosis/en

[16] World Health Organization . CDC Protocol of Realtime RTPCR for Swine Influenza A (H1N1). Geneva, Switzerland: World Health Organization (WHO); 2009.

[17] Chittaganpitch M, Waicharoen S, Yingyong T, et al. Viral etiologies of influenza‐like illness and severe acute respiratory infections in Thailand. Influenza Other Resp. 2018;12(4):482‐489. 10.1111/irv.12554 PMC6005612 29518269

[18] Chuaychoo B, Ngamwongwan S, Kaewnaphan B, et al. Clinical manifestations and outcomes of respiratory syncytial virus infection in adult hospitalized patients. J Clin Virol. 2019;117:103‐108. 31280089 10.1016/j.jcv.2019.07.001 PMC7106545

[19] Bloom‐Feshbach K, Alonso WJ, Charu V, et al. Latitudinal variations in seasonal activity of influenza and respiratory syncytial virus (RSV): a global comparative review. PLoS One. 2013;8(2):e54445. 23457451 10.1371/journal.pone.0054445 PMC3573019

[20] Mehta J, Walsh EE, Mahadevia PJ, Falsey AR. Risk factors for respiratory syncytial virus illness among patients with chronic obstructive pulmonary disease. COPD. 2013;10(3):293‐299. 23536980 10.3109/15412555.2012.744741

[21] Ivey KS, Edwards KM, Talbot HK. Respiratory syncytial virus and associations with cardiovascular disease in adults. J Am Coll Cardiol. 2018;71(14):1574‐1583. 29622165 10.1016/j.jacc.2018.02.013

[22] Pothirat C, Chaiwong W, Phetsuk N, Pisalthanapuna S, Chetsadaphan N, Inchai J. A comparative study of COPD burden between urban vs rural communities in northern Thailand. Int J Chron Obstruct Pulmon Dis. 2015;10(1):1035‐1042. 26082627 10.2147/COPD.S82303 PMC4459631

[23] Thanaviratananich S, Cho S‐H, Ghoshal AG, et al. Burden of respiratory disease in Thailand: results from the APBORD observational study. Medicine (Baltimore). 2016;95(28):e4090. 27428193 10.1097/MD.0000000000004090 PMC4956787

[24] Kitjakrancharoensin P, Yasan K, Hongyantarachai K, et al. Prevalence and risk factors of chronic obstructive pulmonary disease among agriculturists in a rural community, central Thailand. Int J Chron Obstruct Pulmon Dis. 2020;15:2189‐2198. 32982211 10.2147/COPD.S262050 PMC7501975

