# Supplementary material for: Underlying cardiopulmonary conditions as a risk factor for influenza and respiratory syncytial virus infection among community‐dwelling adults aged ≥ 65 years in Thailand: Findings from a two‐year prospective cohort study
Source: Influenza Other Respir Viruses. 2021 Mar 25;15(5):634–40. doi: 10.1111/irv.12855 (PMC8404046; doi:10.1111/irv.12855)
Supplement: Supplementary file 1 — Fig S1 [file IRV-15-634-s001.docx]

Supplemental Figure 1. Incidence of ARI/SARI, RSV, and influenza according to months among participants with and without cardiopulmonary conditions (CPC)
